# Transient enlargement of brain ventricles during relapsing-remitting multiple sclerosis and experimental autoimmune encephalomyelitis

**DOI:** 10.1172/jci.insight.140040

**Published:** 2020-11-05

**Authors:** Jason M. Millward, Paula Ramos Delgado, Alina Smorodchenko, Laura Boehmert, Joao Periquito, Henning M. Reimann, Christian Prinz, Antje Els, Michael Scheel, Judith Bellmann-Strobl, Helmar Waiczies, Jens Wuerfel, Carmen Infante-Duarte, Claudia Chien, Joseph Kuchling, Andreas Pohlmann, Frauke Zipp, Friedemann Paul, Thoralf Niendorf, Sonia Waiczies

**Affiliations:** 1 Experimental Ultrahigh Field Magnetic Resonance, Max Delbrück Center for Molecular Medicine in the Helmholtz Association, Berlin, Germany.; 2 Institute for Medical Immunology, Charité – Universitätsmedizin Berlin, Berlin, Germany.; 3 Medical School Hamburg, University of Applied Sciences and Medical University, Hamburg, Germany.; 4 NeuroCure Clinical Research Center, Charité – Universitätsmedizin Berlin, Berlin, Germany.; 5 Experimental and Clinical Research Center, a joint venture of the Max Delbrück Center for Molecular Medicine and the Charité – Universitätsmedizin Berlin, Berlin, Germany.; 6 MRI.TOOLS GmbH, Berlin, Germany.; 7 Medical Image Analysis Center (MIAC AG) and Department of Biomedical Engineering, University of Basel, Basel, Switzerland.; 8 Department of Neurology, University Medical Center of the Johannes Gutenberg, University of Mainz, Mainz, Germany.

**Keywords:** Autoimmunity, Inflammation, Mouse models, Multiple sclerosis, Neuroimaging

## Abstract

The brain ventricles are part of the fluid compartments bridging the CNS with the periphery. Using MRI, we previously observed a pronounced increase in ventricle volume (VV) in the experimental autoimmune encephalomyelitis (EAE) model of multiple sclerosis (MS). Here, we examined VV changes in EAE and MS patients in longitudinal studies with frequent serial MRI scans. EAE mice underwent serial MRI for up to 2 months, with gadolinium contrast as a proxy of inflammation, confirmed by histopathology. We performed a time-series analysis of clinical and MRI data from a prior clinical trial in which RRMS patients underwent monthly MRI scans over 1 year. VV increased dramatically during preonset EAE, resolving upon clinical remission. VV changes coincided with blood-brain barrier disruption and inflammation. VV was normal at the termination of the experiment, when mice were still symptomatic. The majority of relapsing-remitting MS (RRMS) patients showed dynamic VV fluctuations. Patients with contracting VV had lower disease severity and a shorter duration. These changes demonstrate that VV does not necessarily expand irreversibly in MS but, over short time scales, can expand and contract. Frequent monitoring of VV in patients will be essential to disentangle the disease-related processes driving short-term VV oscillations from persistent expansion resulting from atrophy.

## Abstract 2

Brain ventricle volumes expand and contract during experimental autoimmune encephalomyelitis and relapsing-remitting multiple sclerosis, suggesting that short-term inflammatory processes are interlaced with gradual brain atrophy.

## Graphical abstract

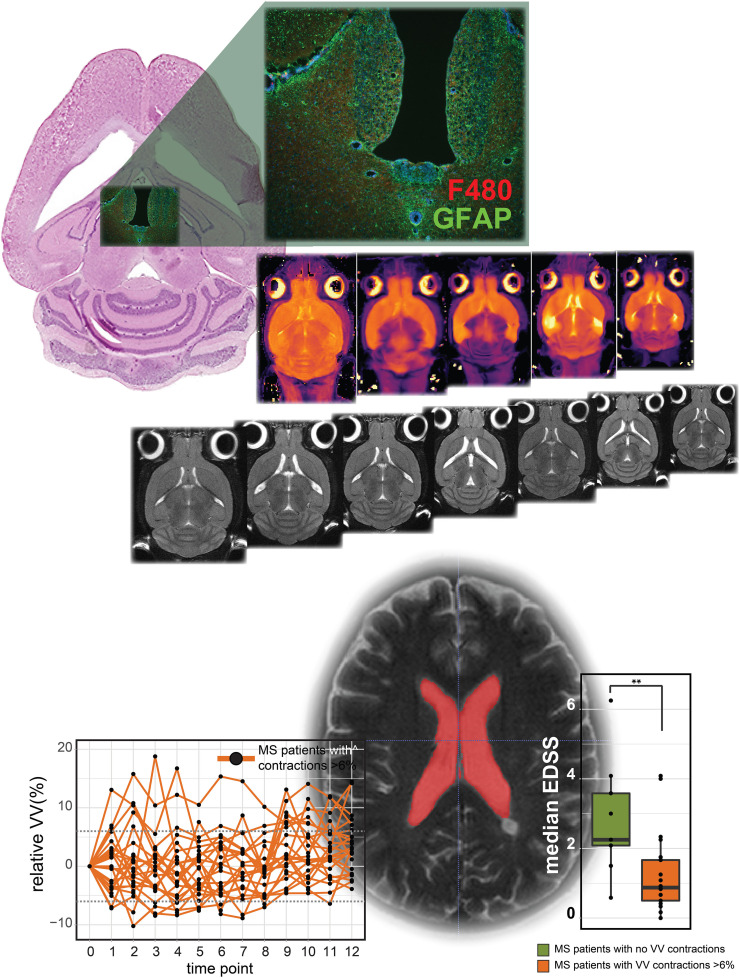

## Introduction

The cerebrospinal fluid (CSF) compartments are increasingly recognized for their vital role in bridging the CNS with the peripheral immune system. Immune cells, including central memory T cells, circulate throughout the CSF in the ventricles and subarachnoid spaces to surveil the CNS parenchyma, after crossing the blood-CSF barrier (BCSFB) in the choroid plexus (1). Together with the blood-brain barrier (BBB), the BCSFB tightly regulates the entry of cells and solutes into the CNS. When these barriers and other regulatory mechanisms break down, immune cells enter the CNS and may initiate neuroinflammatory diseases such as multiple sclerosis (MS) (2). In MS, pathological immune cells trigger demyelination and neurodegeneration, ultimately causing clinical disability.

MRI is an indispensable tool in diagnosing MS and monitoring disease progression (3–6). Disruptions of the BBB can be revealed by MRI using gadolinium-based contrast agents (GBCA). Contrast-enhancing lesions (CEL) indicate recent inflammatory activity, predominantly seen during the relapsing-remitting (RR) disease phases. Contrast enhancement is currently the only MRI feature to assess the lesion acuity at the time of first assessment (7, 8), but increasing concerns regarding potential long-term deposition of gadolinium in the brain could potentially restrict the application of this important diagnostic tool (9–12). Improvements in MRI technology and a better understanding of key aspects of MS pathology promise to better identify changes in the CNS of MS patients and to provide clinicians with a better guide to enhance the accuracy and speed of an MS diagnosis (5).

The experimental autoimmune encephalomyelitis (EAE) animal model of neuroinflammation resembles several aspects of MS and has proven invaluable for gaining insight into its pathological processes and identification of new treatment strategies (13). Studies in the EAE model afford the possibility to directly relate MRI findings with histopathological correlates and to study the phase of disease before clinical onset — investigations that are challenging to perform in patients. We showed, using high-resolution microscopic MRI, that inflammatory lesions could be detected in the cerebellum, cortex, and periventricular regions early in disease, before the onset of clinical EAE signs (14). During this preonset phase of EAE, we observed a marked and reproducible enlargement of the brain ventricles (15). This was surprising, since profound neurodegeneration at this very early stage of disease before the emergence of clinical signs is unlikely to be the explanation for the expansion.

Cerebral ventricle enlargement is commonly linked to neurodegeneration and brain atrophy. While this occurs during the course of normal aging, shrinkage of the cortex and expansion of the ventricles proceeds more rapidly in MS patients (16–21), especially during the secondary progressive phase of MS (SPMS), in which neurodegenerative processes dominate. Nevertheless, neurodegeneration — of the deep gray matter, in particular — can also occur from the earliest stages of brain inflammatory disease (22, 23), and it is essential that aspects of disease relating to inflammation are disentangled from those relating to neurodegeneration. For this, it is crucial that noninvasive investigations are carried out at frequent time intervals in order to capture events that are likely to be overlooked in cross-sectional studies or longitudinal studies with long intervals between observations. Furthermore, correlations among multiple variables during the course of disease should be carefully considered as a time series, to elucidate possible pathological relationships.

To detail fluctuations in the ventricles, the current study examined ventricle volume (VV) changes in both EAE mice and MS patients in longitudinal studies designed with frequent serial MRI scans. We used a time-series approach to study the relationship of VV with other MRI and clinical variables relevant to the pathology. In EAE mice, we performed frequent MRI scans for up to 2 months in order to investigate how inflammatory processes may affect VV beyond the initial acute phase of disease activity. To ascertain the clinical relevance of VV fluctuations in MS, we undertook a retrospective exploratory analysis of data from a prior clinical trial, in which patients with definite RRMS underwent monthly MRI scans over the course of one year. This design afforded a higher time resolution than is typically used, while balancing the practicalities of conducting clinical studies in MS patients. We used time-series analysis to explore how changes in VV over time relate to changes in other MRI and clinical parameters. In this study, we explored the potential of brain ventricle size changes as a possible MRI biomarker that could serve as a proxy of disease activity.

## Results

### VV changes dynamically during the course of EAE.

**Figure 1:**
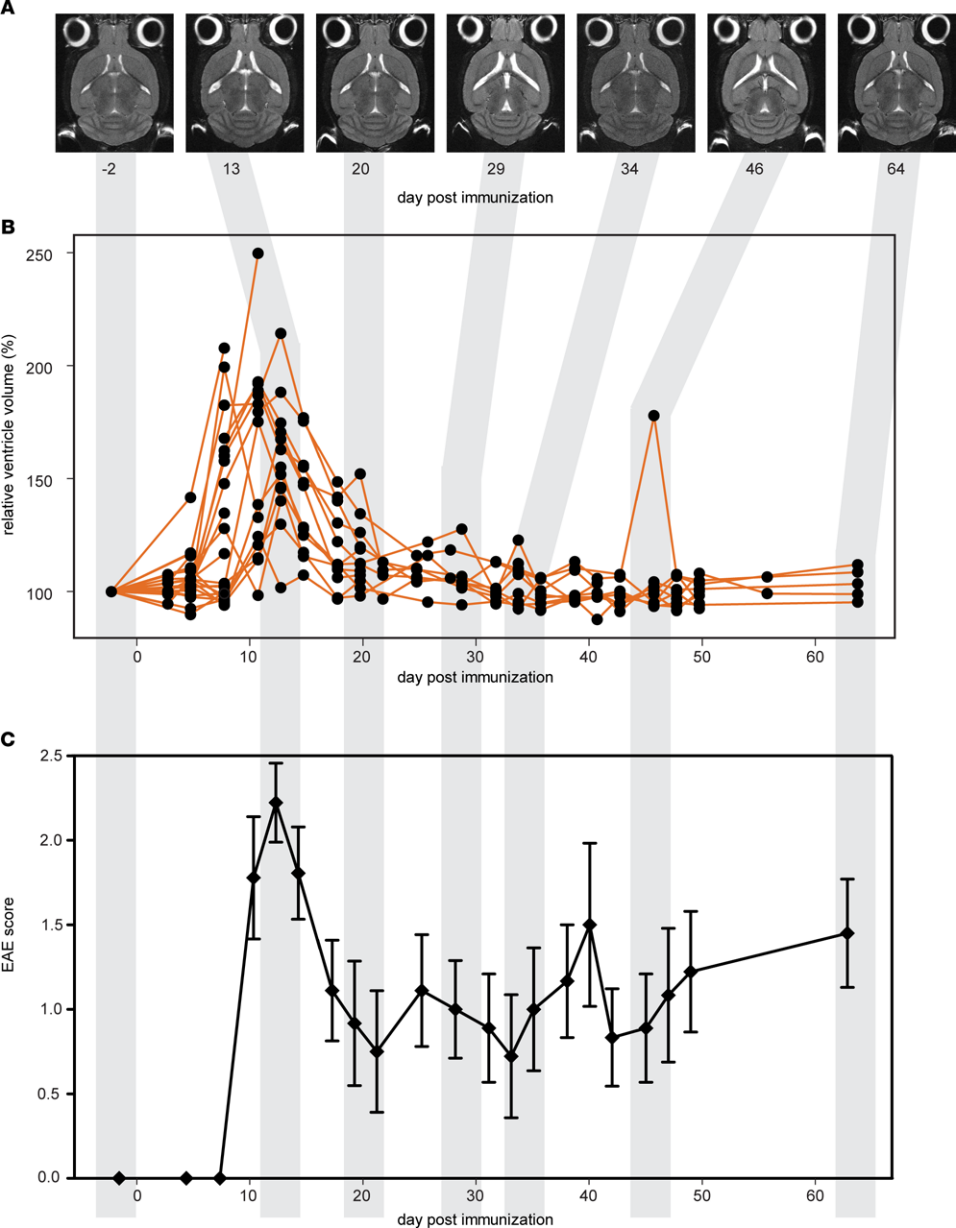
Ventricle volume changes dynamically during the course of EAE. (**A**) T_2_-weighted horizontal MRI scans of a representative mouse brain show changes in ventricle size from baseline (day –2) to day 64 p.i. (**B**) Brain ventricle volume plotted as a percentage of baseline values (*n* = 35). By day 8 p.i., the mean volume was 130.8% ± 7.74% (± SEM) of the baseline volume (*P* < 0.001, ANOVA), well beyond the range of normal variation in healthy SJL mice of ± 6%. Ventricle volume peaked at days 11–13 p.i. and returned to baseline levels by day 22 p.i. (**C**) Emergence and remission of EAE clinical signs coincided with the peak expansion and contraction of ventricle volume (mean ± SEM).

We performed frequent MRI of EAE mice over a period of 2 months in order to investigate changes beyond the presymptomatic phase and the first disease peak and remission, typically examined in many EAE studies. During the initial disease phase, there was a pronounced increase in VV that is clearly visible on anatomical MRIs (Figure 1A), confirming our previous observation (15). By day 8 (d8) postimmunization (p.i.), the group mean increase in VV relative to baseline values was 130.8% ± 7.74% (mean ± SEM, *P* < 0.001, 1-way ANOVA) (Figure 1B). This was well beyond the range of normal VV variation in healthy unmanipulated SJL mice, which we determined in our previous study (*n* = 6) to be plus or minus 6% (15). This increase peaked at 160.7% ± 10.25% and 157.5% ± 7.69% by d11 and d13 p.i. Four mice in the cohort showed VV increases greater than 200%.

The magnitude of expansion began tapering at d15 p.i. (139.9% ± 6.68%), and mean VV eventually returned to baseline levels by d22 (Figure 1B). For *n* = 9 SJL mice, we continued MRI examinations every 2–3 days, up to d64 p.i., in order to follow the VV changes downstream of the initial disease phase. The mice showed a typical RR disease course, with clinical signs developing around d10 p.i., peaking at d14 p.i., and remitting by d20–d22 p.i. (Figure 1C).

The expansion and contraction in VV followed a similar course as the neurological signs. In the representative case shown in Figure 1A, VV expansions can be seen at d13, d29, and d46 p.i., each followed by VV contractions at d20, d34, and d64, respectively (Figure 1A). The timing of VV normalization coincided with remission of clinical signs, although there were differences in the kinetics among individual animals, especially at later time points. VV continued to change during the chronic stage of disease, though generally, these fluctuations were less pronounced than the prominent changes observed during the initial acute disease phase. All animals that were monitored after the initial VV expansion showed contractions back to normal volumes — i.e., no animal displayed permanently enlarged ventricles. Importantly, at the end of the experiment (d64), while most animals still exhibited a degree of neurological disease severity (EAE score 2.0 ± 1.75, median ± IQR), VVs had returned to normal baseline values.

### Ventricle enlargement coincides with emergence of gadolinium-enhancing lesions and precedes onset of clinical signs of EAE.

To explore the relationship between VV changes and BBB breakdown during inflammation, we administered GBCA to EAE mice during the early acute phase of disease. T_1_ mapping was performed to obtain an unbiased quantitative measurement of the global changes in tissue relaxation caused by leakage of the GBCA into the brain parenchyma. In order to make the quantification robust across the longitudinal series of scans, images from each time point were registered to the baseline image (2 days before immunization [d–2]), for each individual animal.

**Figure 2:**
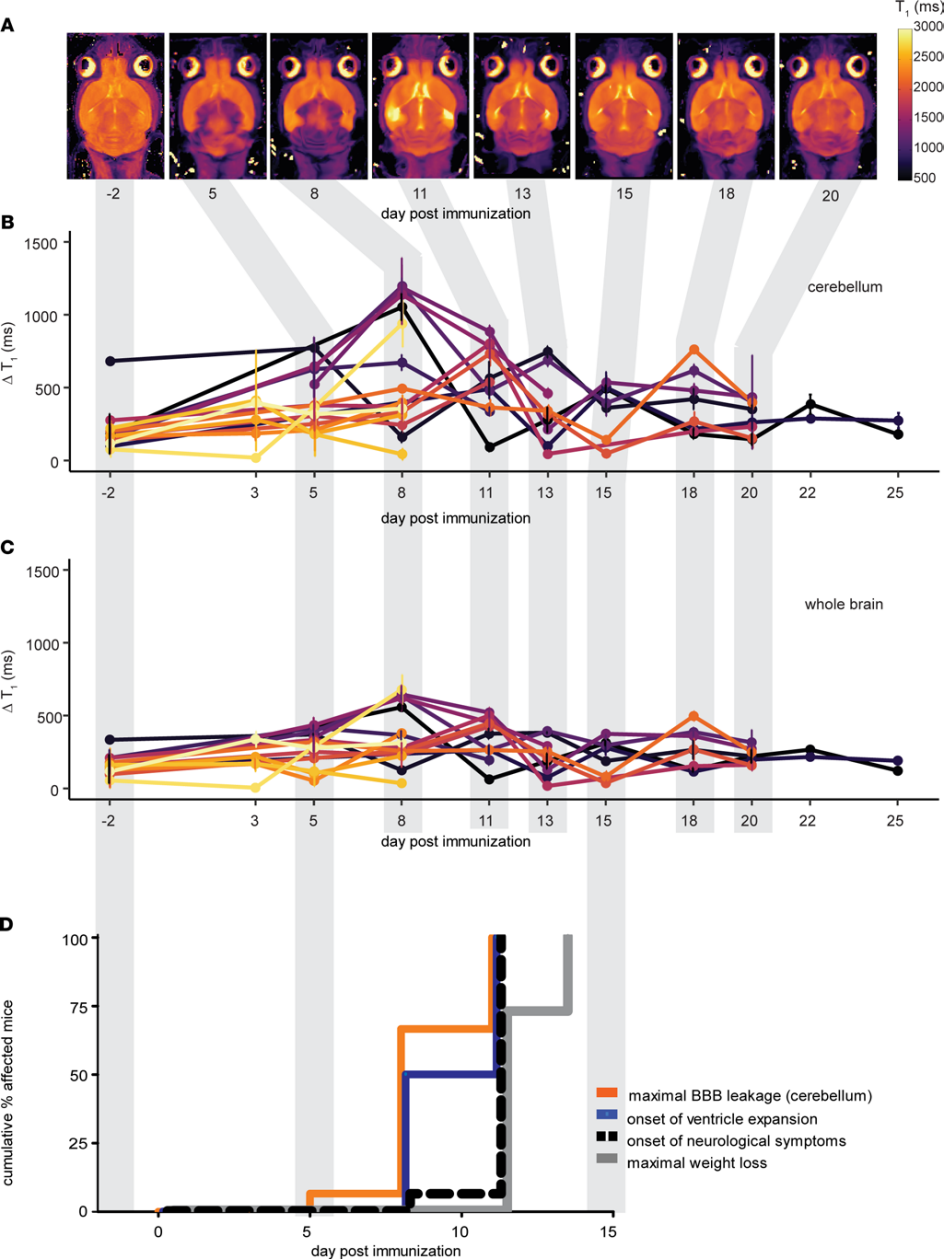
Ventricle enlargement coincides with gadolinium-enhancing lesions and precedes EAE clinical signs. (**A**) T_1_ map MRIs of a representative mouse brain show altered tissue relaxation due to blood-brain barrier disruption following administration of gadolinium-based contrast agent. Reduced tissue T_1_ (purple) is apparent in the meninges, cerebellum, and periventricular regions already by day 5 p.i. Note that the brain images have been registered to the baseline image for quantification; therefore, changes in ventricle volume are not apparent in these images. (**B**) Quantification of global changes in tissue T_1_ following gadolinium contrast administration were especially prominent in the cerebellum (*n* = 16). The ΔT_1_ (precontrast – postcontrast values) was significantly increased from baseline at days 8–11 p.i. (*P* = 0.0030, ANOVA). (**C**) The ΔT_1_ of the whole-brain was also significantly increased at days 8–11 p.i. (*P* = 0.0023, ANOVA). (**D**) Kaplan-Meier plots show that the time of onset of ventricle expansion and the time of maximal gadolinium enhancement significantly preceded the onset of EAE clinical signs (*P* = 0.0068 and *P* = 0.0005, respectively; log-rank test) and the time of maximal body weight loss (*P* = 0.008, *P* < 0.0001, respectively).

A typical pattern of contrast enhancement — with diffuse lesions especially prominent in the cerebellum, meninges, and periventricular regions — was observed already by d5 p.i. (Figure 2A). The change in brain T_1_ values after GBCA administration (ΔT_1_ = precontrast minus postcontrast mean values) was calculated for *n* = 16 mice, at time points ranging from baseline (d–2 p.i.) to d25 p.i. (Figure 2, B and C). Compared with baseline, the ΔT_1_ in the cerebellum was significantly increased at d8 and d11 p.i. (592.9 ± 103.8 ms and 551.2 ± 71.4 ms, respectively, *P* = 0.0030, 1-way ANOVA) (Figure 2B). The ΔT_1_ in the whole brain (Figure 2C), was also significantly increased at d8 and d11 p.i. (373.1 ± 52.9 ms and 342.8 ± 43.2 ms, respectively, *P* = 0.0023, 1-way ANOVA).

Gadolinium enhancement was observed before the onset of clinical signs (Figure 2D), although the timing of this varied among animals. We compared the timing of changes in ΔT_1_ with that of VV and clinical changes (Figure 2D). There was no significant difference in the time to show VV increases > 6% and the time to show the maximum change in ΔT_1_ (*P* = 0.2800, log-rank test) (Figure 2D). Both VV and ΔT_1_ increases occurred significantly earlier than the onset of EAE clinical signs (*P* = 0.0068 and *P* = 0.0005, respectively). The onset of EAE clinical signs is often accompanied by a substantial reduction in body weight. Maximum body weight loss also occurred significantly later than both VV and ΔT_1_ changes (*P* = 0.008 and *P* < 0.0001, respectively) (Figure 2D).

### Early ventricle enlargement in EAE correlates with inflammation and minimal neurodegeneration.

**Figure 3:**
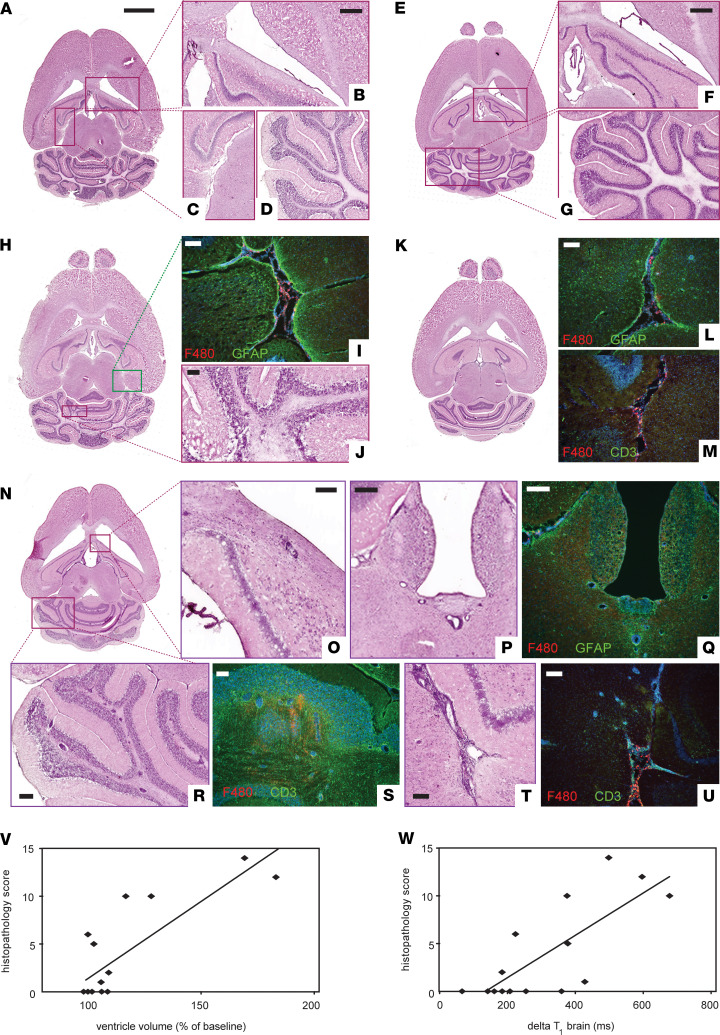
Early ventricle enlargement in EAE correlates with inflammation. (**A**–**G**) Representative images of H&E-stained tissue sections show absence of pathology at baseline (**A**–**D**) and day 3 p.i. (**E**–**G**). Higher magnifications views show periventricular (**B** and **F**), meningeal (**C**), and cerebellar regions (**D** and **G**). (**H**–**J**) First signs of pathology were detected on day 5 p.i., as infiltration of macrophage/myeloid cells (F4/80^+^, red) and accompanying gliosis (GFAP, green) in meningeal areas (**I**) and inflammatory foci in cerebellar white matter (**J**). (**K**–**M**) By day 8 p.i., pronounced meningeal infiltration and gliosis was present (**L**), along with T cell infiltration in the parenchyma (CD3^+^, green) (**M**). (**N**) At day 11 p.i., ventricular enlargement was grossly apparent from the whole brain tissue sections. (**O**–**U**) Extensive inflammation was seen throughout the brain, including periventricular regions (**O**, **P**, **Q**), cerebellum (**R**, **S**), and meningeal areas (**T**, **U**), along with gliosis (**Q**) and infiltration of F4/80 and CD3 positive cells (**Q**, **S**, **U**). (**V** and **W**) Semiquantitative scoring of histopathology (*n* = 19) correlated positively with ventricle volume changes (**V**) and gadolinium enhancement as ΔT1 changes in whole brain (**W**) (Spearman’s *r*). Scale bars: **A** (whole brain images): 2 mm; **B**–**D**, **F**, and **G**: 500 μm; **P**–**R**: 200 μm; **I** and **J**, **L** and **M**, **O**, and **S**–**U**: 100 μm.

The macroscopic VV expansion observed by MRI could also be seen by gross examination of histological sections (Figure 3). With histological staining, we could follow the emergence of inflammatory cell infiltration in EAE brains (*n* = 19) up to d11 p.i. (Figure 3, A–U). Representative images of H&E-stained sections show no apparent pathology before immunization (Figure 3, A–D) or at d3 p.i. (Figure 3, E–G). The first signs of pathology were detected on d5 (Figure 3, H–J), as shown by the start of meningeal infiltration of macrophage/myeloid cells (F4/80 staining) and accompanying gliosis (GFAP staining) (Figure 3I) and the appearance of cerebellar lesions (Figure 3J). By d8 p.i., the inflammation was even more evident (Figure 3, K–M). This corresponds to the time point of statistically significant increases in VV and GBCA enhancement observed by MRI. At this time point, we observed pronounced meningeal inflammation and gliosis (Figure 3L), as well as T cell infiltration in the parenchyma, as shown by CD3 stains (Figure 3M). By d11 p.i., the marked increase in volume of the lateral, third, and fourth ventricles (Figure 3N) was accompanied by extensive histopathology, especially prominent in periventricular regions (Figure 3, O–Q) and cerebellum (Figure 3, R and S), as well as brainstem and meninges (Figure 3, T and U).

Semiquantitative scoring of the severity of inflammation positively correlated with VV — i.e., mice with the largest increase of VV showed greater burden of histopathology (Spearman’s *r* = 0.7225, *P* = 0.0023) (Figure 3V). The histopathology score also correlated with the ΔT_1_ of the brain (Spearman’s *r* = 0.7480, *P* = 0.0013) (Figure 3W).

**Figure 4:**
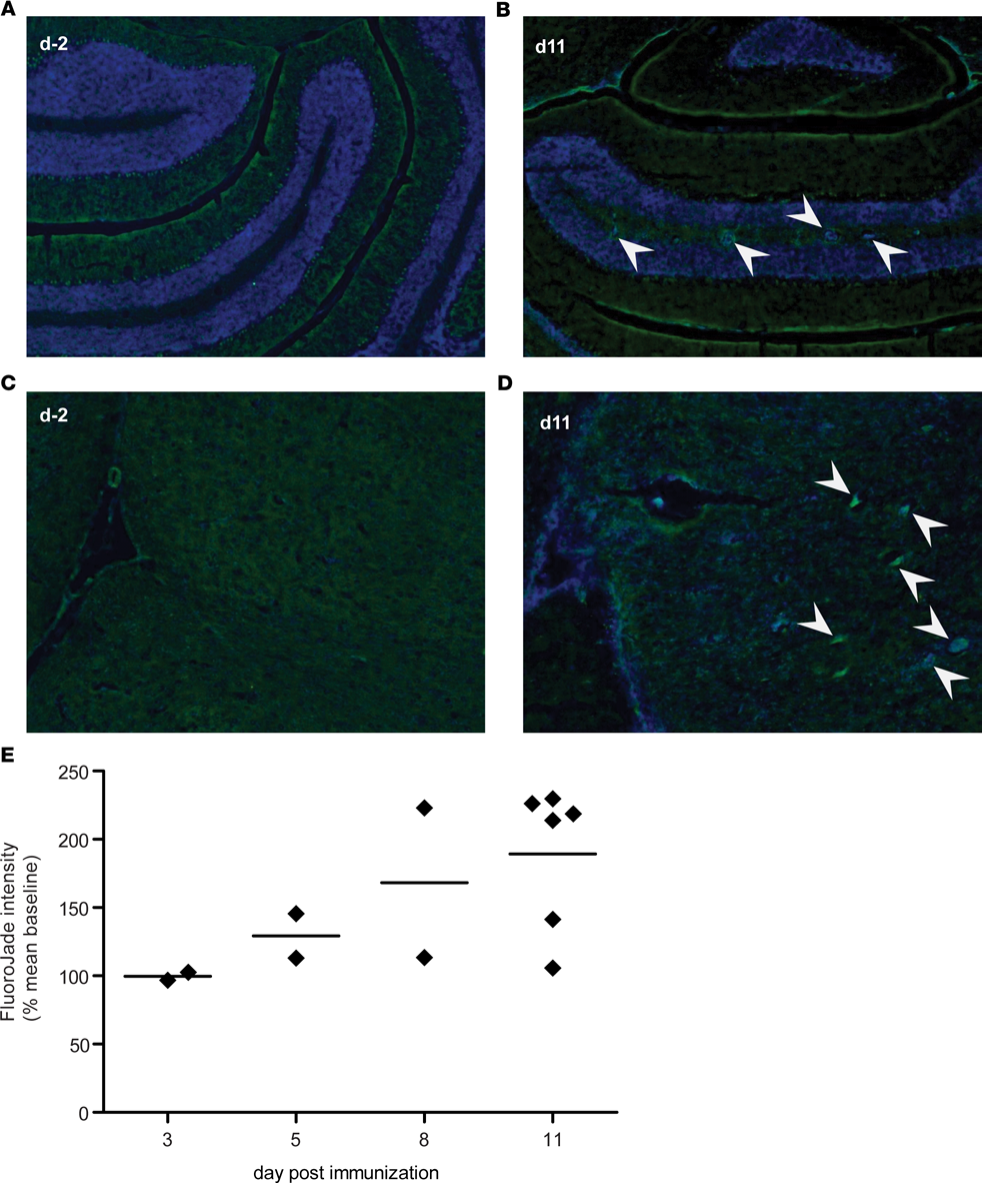
Modest neurodegeneration detected by Fluoro-Jade staining during early EAE. (**A** and **C**) No evidence of Fluoro-Jade^+^ staining was seen in brain tissue sections from unimmunized control mice; representative images of cerebellum (**A**) and brainstem (**C**). (**B** and **D**) Fluoro-Jade^+^ foci (indicated with arrows) were observed in cerebellum (**B**) and brainstem (**D**) of mice at day 11 p.i. (Fluoro-Jade, green; DAPI, blue). The Fluoro-Jade^+^ foci consistently accompanied inflammatory foci (**B** and **D**). (**E**) Quantification of fluorescence intensity of the tissue section showed an increasing accumulation of Fluoro-Jade staining by day 11 p.i. (arbitrary units) (*n* = 15).

Neurodegeneration is expected during the course of EAE. We performed Fluoro-Jade (FJ) staining on serial sections from the same tissue samples shown in Figure 3 in order to determine to what extent this related to the inflammatory pathology and VV enlargement. No FJ^+^ staining was seen in unimmunized animals (Figure 4, A and C) or at d3 p.i. FJ^+^ foci were seen infrequently on d5 p.i., became more readily apparent by d8 p.i., and were more frequently observed by d11, in cerebellum and the brainstem (Figure 4, B and D). Although FJ^+^ foci always accompanied inflammatory lesions, only minimal FJ staining was observed in periventricular lesions. Quantification of FJ staining intensity from whole brain sections illustrates a modest yet increasing accumulation of neurodegeneration during this early phase of EAE (Figure 4E).

### Dynamic changes in VVs in RRMS patients.

**Figure 5:**
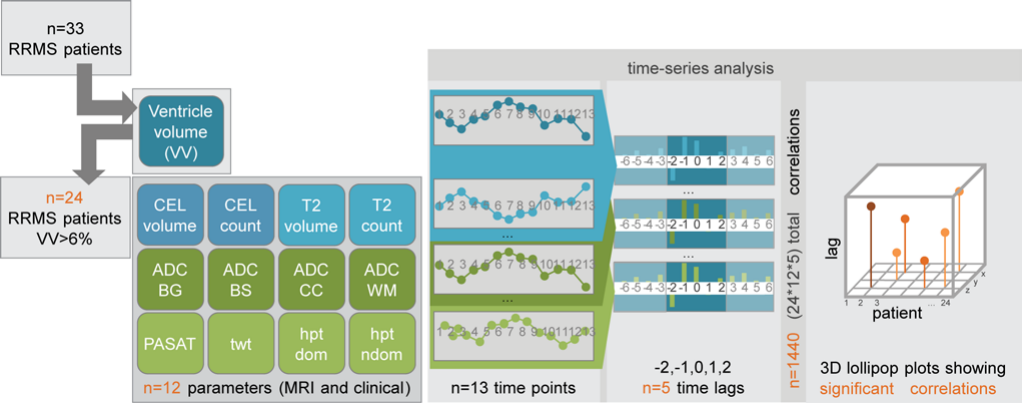
Schematic for time-series analysis workflow. From the cohort of *n* = 33 RRMS patients, we performed the time-series analysis on the subset of *n* = 24 patients who showed contractions in ventricle volume greater than the ± 6% range of normal variation. Ventricle volumes were measured at 13 monthly time points. At the same time points, an additional 8 MRI parameters and 4 clinical parameters were measured. This allowed each of these measures to be considered as a time series. Using the cross-correlation function, the cross-correlation coefficients between 2 time series can be calculated; significant coefficients indicate that events of one series precede (negative time lag) or follow (positive time lag) the events of another series. In the current study, we limited the consideration of significant cross-correlation coefficients to ± 2 time lags (i.e., ± 2 months). From *n* = 24 patients, *n* = 12 variables, and *n* = 5 time lags (including the 0 time lag), a total of 1440 coefficients was calculated. From the coefficients with nominal *P* < 0.05, the FDR correction for multiple comparisons was applied, to yield the corrected significant cross-correlation coefficients, which were then displayed in the 3D plots.

The magnitude and timing of the VV changes in the EAE model were compelling. Nevertheless, the validation that these findings in the animal had any translational relevance for MS patients remained to be demonstrated. For this, we performed a time-series analysis (Figure 5) of data from our previous clinical study (NCT00616187) (24), in which RRMS patients (*n* = 33) underwent 13 serial MRI examinations over 1 year (Figure 5). This was a baseline-to-treatment clinical study of oral high-dose atorvastatin treatment for RRMS; all patients received atorvastatin, and 10 of 33 patients also received IFN-β throughout the study. This study included both MRI (*n* = 8) as well as clinical (*n* = 4) parameters measured at each time point.

**Figure 6:**
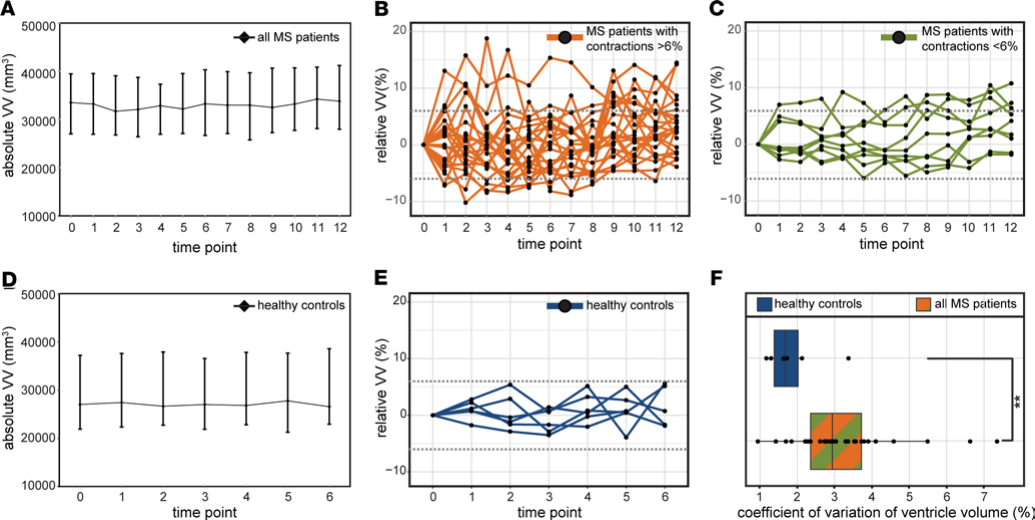
Relapsing-remitting MS patients show dynamic changes in ventricle volumes. (**A**) Over the course of 1 year, the cohort of MS patients showed a small but significant increase in ventricle volume of +284 mm, equivalent to +0.08406% (median ± IQR, *P* = 0.0006, Wilcoxon signed rank test, *n* = 33). (**B** and **C**) Plotting the values for individual patients showed a heterogeneous picture, with some individuals showing high variability in ventricle volume (**B**) and others showing lower variability (**C**). (**D**) A reference cohort of healthy subjects showed no significant changes over the course of a 6-month observation period (*n* = 6). (**E**) The maximum change in ventricle volume of the healthy subjects was ± 6%. (**F**) The MS patient cohort showed significantly greater variability in ventricle volumes (as indicated by the coefficient of variation) compared with healthy controls (**P = 0.0065, Student’s *t* test).

Considering the entire cohort of 33 patients, there was a small but significant increase in the median VV between baseline and the last time point at the conclusion of the study: baseline volume = 33,500 mm^3^ (27,028 – 39,468) (median ± IQR) versus 33,784 (27,953 – 41,185), resulting in an increase of 284 mm^3^, equivalent to an increase of 0.08406% (*P* = 0.0006, Wilcoxon’s signed rank test) (Figure 6A). However, plotting the VV changes over time, expressed as percent changes relative to the VV at baseline for each individual, revealed a highly heterogeneous picture. Some patients showed substantial volatility in VV over time (Figure 6B), while others showed minimal changes (Figure 6C). To distinguish among cases with varying VV volatility, we first determined the normally expected range of intraindividual variation in VV in healthy individuals by making use of data from a study that performed serial MRI of healthy subjects over 6 months (25), selecting time points to match the 1-month time intervals separating scans of the MS cohort. We calculated the median VV for each time point (Figure 6D) and changes in VV relative to baseline for each healthy control (Figure 6E). From this data, we determined that the intraindividual variation in VV fluctuated by plus or minus 6% over time in healthy subjects (Figure 6E). This result was corroborated by analysis of data from a second study that performed serial scans on healthy individuals (26).

In a large proportion of MS patients (21 of 33, 64%), changes in VV exceeding the 6% threshold were observed within 1-month intervals. A subset of patients (5 of 33, 15%) showed exclusively positive changes in VV over the duration of the study — but no contractions. When this minority was excluded from the analysis, the remaining patients showed a small overall decrease in VV (33,000 [27,560 – 39,750] versus 32798 [28,323 – 41,490], –0.00612% [baseline versus last time point, median ± IQR, *P* = 0.0098, Wilcoxon’s]). A larger fraction of patients, 24 of 33 (73%), showed VV contractions beyond the 6% threshold, which extended consecutively over a period of at least 2 months, with no expansions in between. VV changes in this cohort were not a monotonic increase in volume throughout the study period. Based on this threshold, we segregated the patients into 2 groups, considering that a VV contraction event (beyond the level of normal variation) would necessarily follow an expansion event and that such a contraction could exclude VV changes due to brain atrophy. One group (*n* = 24) was labeled as MS patients with contractions > 6% (Figure 6B), and the other group (*n* = 9) was labeled as noncontracting MS patients (Figure 6C). The range of plus or minus 6% is depicted by a dotted line (Figure 6, B, C, and E). The whole MS patient cohort had significantly greater volatility in ventricle size, with the coefficient of variation (CV) in VV of 3.235 ± 0.234 versus that of controls 1.887 ± 0.327 (mean ± SEM, *P* = 0.0065, 2-tailed Student’s *t* test) (Figure 6F).

**Figure 7:**
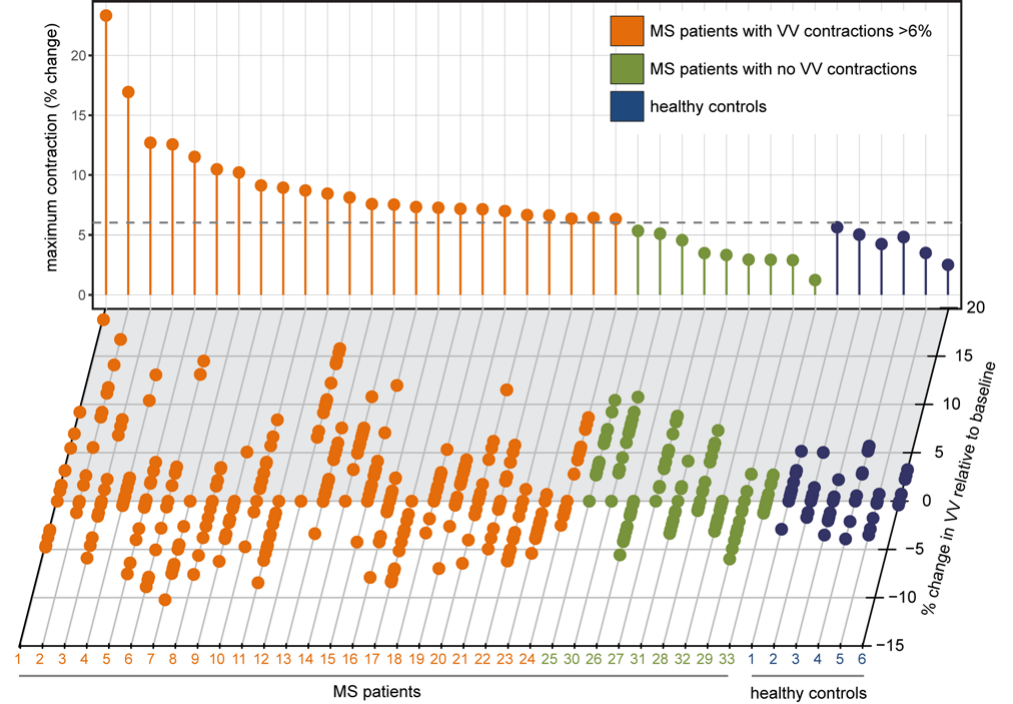
MS patients show variable ventricle volume (VV) changes. Individual MS patients showed considerable variability in the patterns of VV changes during the course of 13 monthly MRI examinations. Ventricle volumes from each patient and healthy subject are shown along the *x* axis; each dot represents 1 time point. Results are depicted as a percentage change from the baseline measurement. The vertical lollipop lines indicate the maximum contraction observed for each individual during the study period, in percentage change. The dotted line illustrates the maximum ventricle contraction observed in the healthy cohort: 6%. Based on this threshold, the cohort was divided into MS patients with contractions > 6% (orange) and MS patients without contractions (green). Healthy subjects are depicted in blue.

Even within each patient group, there was a large variation in the magnitude of VV changes, as shown from the plot of relative VV changes for each individual patient during the study (Figure 7, lower panel). The maximum VV contraction for each patient (over 2 months) is depicted in the lollipop plot (Figure 7, upper panel).

**Figure 8:**
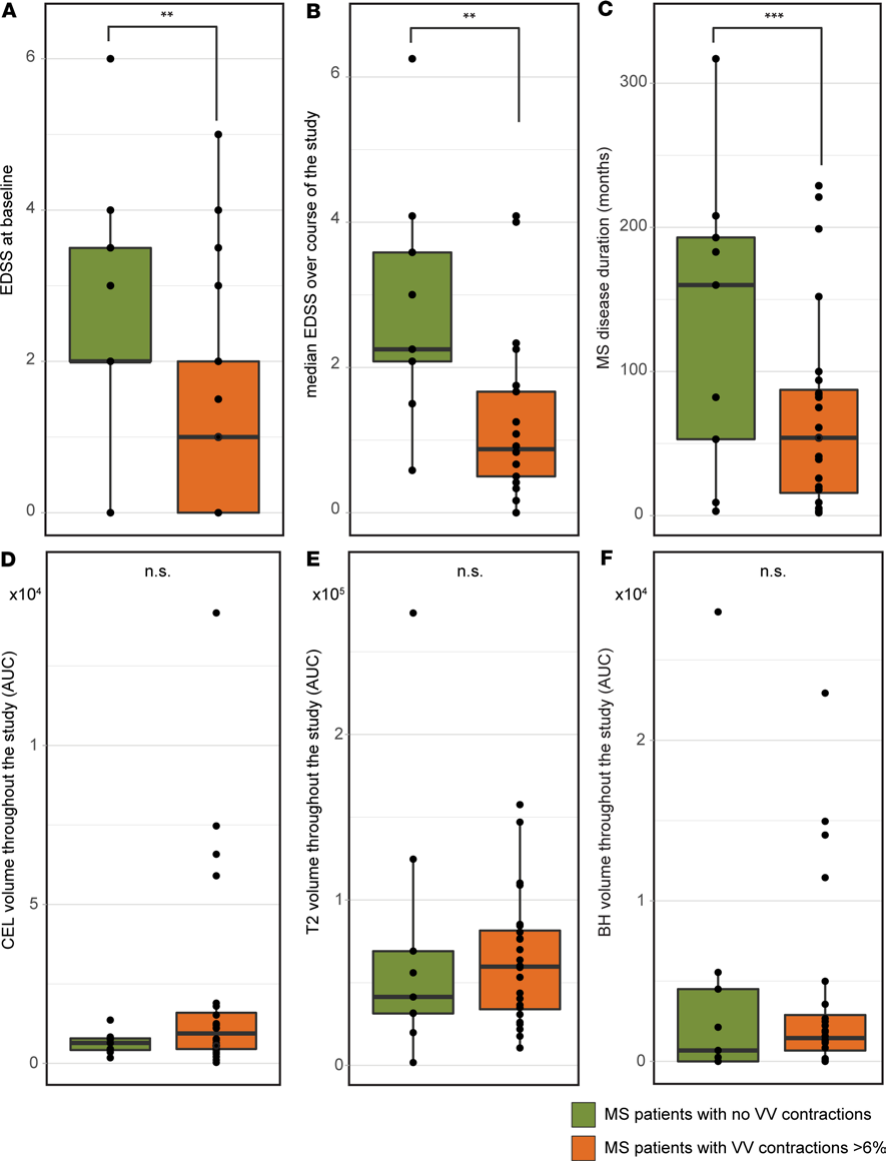
MS patients with contracting ventricles have lower disease severity and duration. (**A** and **B**) MS patients who showed contractions (*n* = 24) in ventricle volumes greater than 6% had significantly lower disease severity at the study baseline, as indicated by the Expanded Disability Status Scale, EDSS) (**P = 0.0221, Mann-Whitney test) (**A**), and significantly lower median EDSS during the study period (**P = 0.0063, Mann-Whitney test) (**B**). (**C**) The patients with ventricle contractions also had significantly shorter total disease duration (***P = 0.046, Student’s *t* test). (**D**–**F**) There were no significant differences between contracting and noncontracting patients (*n* = 9) in volume of CEL (**D**), T_2_ (**E**), or BH lesions (**F**) over the course of the study.

Upon stratifying the patients, we investigated potential differences between the groups that could be relevant to MS. MS patients with VV contractions > 6% had significantly lower baseline expanded disability status scale (EDSS) and median EDSS throughout the study period compared with patients with no VV contractions > 6% (*P* = 0.0221, *P* = 0.0063, respectively; Mann-Whitney *U* test) (Figure 8, A and B). Patients with contracting VV also had a significantly lower total disease duration time (*P* = 0.046, 2-tailed Student’s *t* test) (Figure 8C). There were no significant differences between contracting and noncontracting patients in the total burden of CEL, T_2_, or black hole (BH) lesion volume over the course of the study (*P* = 0.289, *P* = 0.592, *P* = 0.598, respectively; Mann-Whitney *U* test) (Figure 8, D–F). Contracting and noncontracting patients did not differ in terms of age, sex, the proportion also receiving IFN-β therapy, or number of acute relapses during the study period (*P* = 0.26, *P* = 0.748, *P* = 0.511, *P* = 0.582, respectively; 2-tailed Student’s *t* test, χ^2^ test for proportion with therapy). Patients did not show any significant loss of total brain volume (BV) during the course of the study, either when considering the entire cohort (*P* = 0.1167) or the contracting and noncontracting patients separately (*P* = 0.8750, *P* = 0.9770, respectively; 2-way ANOVA with Tukey’s post hoc test; Supplemental Figure 1; supplemental material available online with this article; https://doi.org/10.1172/jci.insight.140040DS1). Even when comparing across all time points, we did not observe significant changes in BV. There was, however, a significant negative correlation between VV and overall BV, considering the entire cohort, at selected time points (Supplemental Figure1 and Supplemental Table 2).

### Time-series analysis of VV changes in MS.

Given the heterogeneity of VV changes in the MS cohort, analysis of group means can potentially obscure relationships between VV changes and other parameters that are relevant to MS pathology. To address whether significant temporal relationships exist between VV changes and changes in other MRI and clinical parameters, we used time-series analysis (a schematic outline of the workflow is depicted in Figure 5). All clinical and MRI parameters (including VV measured from anatomical T_1_ and T_2_ images) acquired at each time point during the study were included in the analysis. Given the parametric nature of the time-series analysis, ordinal variables such as EDSS were not included. We performed the time-series analysis on the subset of 24 MS patients who showed VV contractions above the 6% threshold.

**Figure 9:**
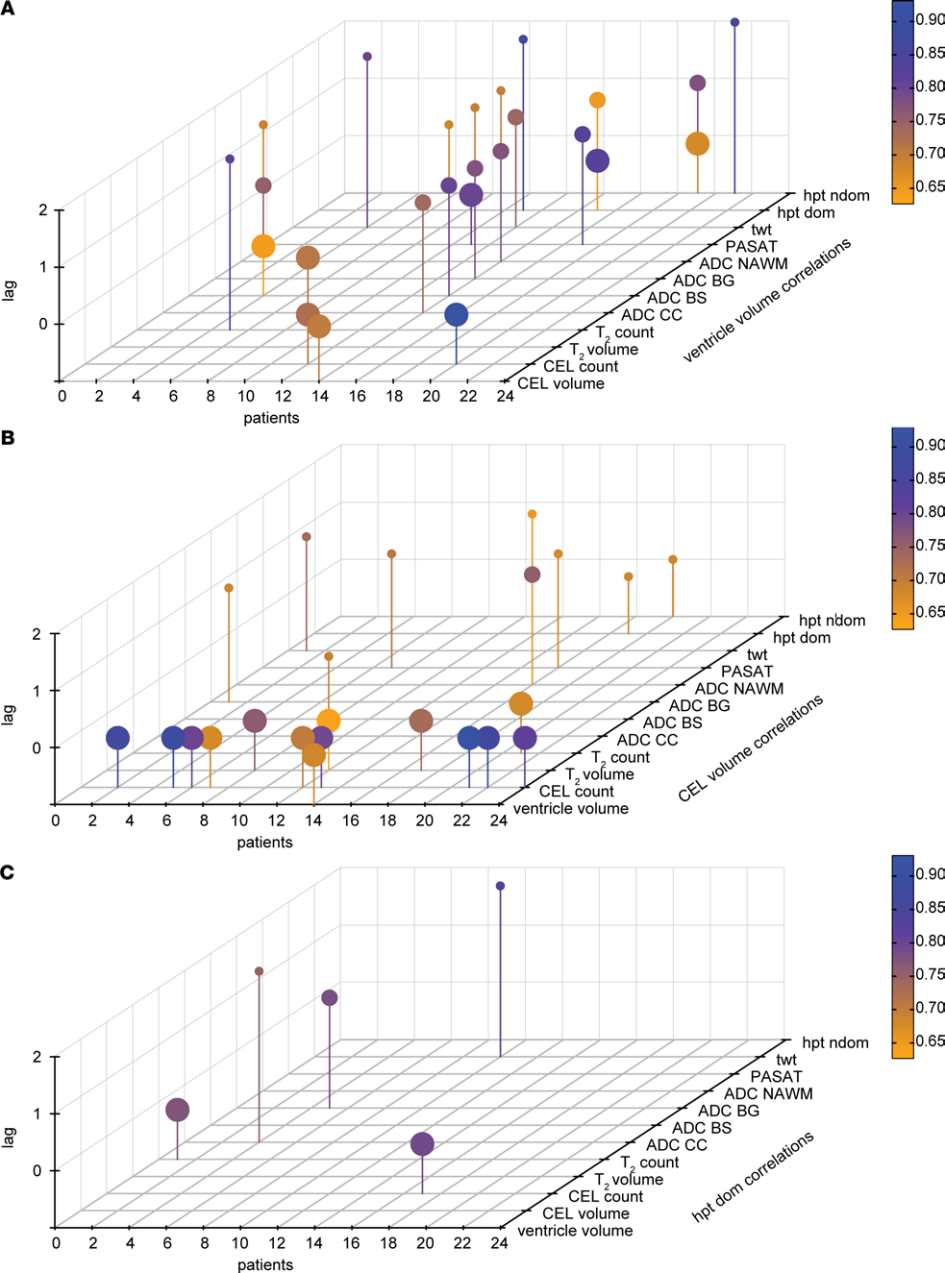
Time-series analysis of ventricle volume changes in MS. (**A**) For each individual patient with ventricle volume contractions > 6% (*n* = 24), we calculated the cross-correlation function (CCF) between the time series of ventricle volume (as the X variable) and the time series of the other 8 MRI and 4 clinical measures (as the Y variables). The CCF yielded 25 significant cross-correlation coefficients, within a time lag of ± 2 months, after FDR correction for multiple comparisons. The significant coefficients are depicted as lollipops in the 3D plot; color is scaled to the magnitude of the coefficient. Individual patients are arranged on the *x* axis; the MRI and clinical parameters are arranged on the *y* axis. The absolute values of the time lags are shown on the *z* axis, and size of the lollipop is scaled to the time lag (lag 0, largest). (**B**) Using the time series of CEL volume as the X variable and the other MRI and clinical parameters (including ventricle volume) as the Y variables, the CCF analysis yielded 23 significant cross-correlation coefficients after correction for multiple comparisons. Of these, 9 of 23 were correlations between CEL volume and CEL count at 0 time lags. (**C**) Repeating the analysis using the time series of performance in the 9-hole peg test as the X variable yielded 5 significant cross correlation coefficients after correction for multiple comparisons.

We calculated the cross-correlation function (CCF) for each individual patient using VV as the X variable and the other 8 MRI and 4 clinical parameters as Y variables. We considered only those significant cross-correlation coefficients that occurred within plus or minus 2 time lags (i.e., ±2 months) as reflecting a meaningful temporal relationship with VV changes (Figure 9A). The CCF analysis resulted in a total of 101 cross-correlation coefficients with nominal *P* < 0.05. Upon FDR correction for multiple comparisons, 25 significant coefficients were retained (Figure 9A). The significant cross-correlation coefficients were detected in 10 of 24 MS patients included in the analysis. These were distributed among all clinical parameters and most of the MRI (7 of 8) parameters. All 5 time lags considered (0, ±1, and ±2 months) appeared among the significant cross-correlations.

Detection of CEL is a major criterion for MS diagnosis. The CCF analysis was repeated using CEL volume as the X variable and the remaining 12 parameters (including VV) as Y variables (Figure 9B). This analysis yielded a total of 96 cross-correlations with nominal *P* < 0.05. This was reduced to 23 significant cross-correlations following FDR correction for multiple comparisons (Figure 9B). Of note, 9 of 23 significant cross-correlations were correlations between CEL volume and CEL count, which was to be expected, and 14 of 23 were at 0 lags (i.e., the changes occurred at the same time as CEL volume changes).

As an additional comparison, we repeated the CCF analysis using the performance in the 9-hole peg test of the dominant hand (hpt dom) as the X variable and comparing it with the other 12 parameters (including VV) as Y parameters (Figure 9C). This analysis yielded 73 cross-correlations with nominal *P* < 0.05; only 5 significant cross-correlations were retained following correction for multiple comparisons (Figure 9C).

## Discussion

In this study, we demonstrate a pronounced enlargement of the mouse brain ventricles of up to 200%, before the onset of clinical signs, during the first disease peak in EAE. We show that this enlargement resolves at the time of clinical remission, strongly suggesting that it is not the result of brain atrophy due to neurodegeneration. The VV continues to fluctuate during the disease course. This phenomenon has clinical relevance, as the majority of RRMS patients in our cohort showed dynamic enlargement and contraction of VV, beyond the range of variation seen in healthy individuals. Those patients who showed the most volatility in VV appeared to be at an earlier stage of disease; they had significantly lower baseline and median EDSS throughout the study period, and they had a significantly shorter total disease duration compared with the patients who did not show contractions. Crucially, these results demonstrate that VV in RRMS patients does not only increase unidirectionally over time as a result of atrophy, but in the majority of cases, VV expands and contracts dynamically during the disease course. These short-term fluctuations occur at a time scale of less than 1 year and are unlikely to be the result of neurodegeneration exclusively. They suggest that other disease-related processes might be contributing factors. Close monitoring of VV changes during disease could therefore offer further valuable insights.

A distinguishing feature of this work is the relatively high time resolution of the longitudinal studies. EAE mice were scanned every 2–3 days over a period of 64 days. EAE studies typically consider fewer time points during the disease course. Long intervals between observations can limit the detection of potential changes that take place at shorter time scales, obscuring the complexities of the underlying processes. Similarly, the clinical study we investigated for time-series analysis was originally designed to evaluate the efficacy of atorvastatin in RRMS patients and included 13 monthly MRI investigations over a period of 1 year. These time intervals are shorter than those typically considered in clinical practice and offer the possibility to capture subtle changes that might otherwise remain unnoticed.

In our analysis, we considered changes at the individual patient level. When analyzing the MS patients as a group, we observed a small though significant increase in VV between the start and end of the study, which is consistent with the conventional interpretation of neurodegeneration and brain atrophy (27). However, there was no significant change in BV throughout the study, suggesting that subtle atrophy may be proceeding on a central rather than global level (28, 29). It is expected that VV will increase over time in the course of normal aging, and it is indisputable that this is accelerated in MS (19, 21, 30, 31). When studying the VV in MS patients individually, we observed an oscillating behavior in the majority of patients, with VV expansions and contractions. This suggested that, superimposed upon the long-term expansion of VV resulting from neurodegeneration, other disease-related processes drive the short-term oscillations in VV. Another confounding process is the phenomenon of pseudoatrophy (nontissue related BV reduction) that can occur following introduction of antiinflammatory therapies (32–34) as a result of accelerated water losses and fluid shifts (35), as well as resolution of immune cell infiltration. The patients in the clinical trial that we retrospectively investigated were treated with atorvastatin (and some also with IFN-β) but did not show any signs suggestive of pseudoatrophy (24). During the relatively short duration of our study (1 year), there was no significant reduction in BV in all cohorts. Aside from pseudoatrophy, some immunomodulatory agents (e.g., S1P agonists) appeared to slow down BV losses. In addition to reducing new MRI activity, 6 months of treatment with fingolimod resulted in significantly lower BV losses compared with placebo control (36), suggesting neuroprotective mechanisms were operating, beyond the expected antiinflammatory activity. In parallel to monitoring BV changes, it would be interesting to follow changes in VV in S1P agonist–treated patients as a possible surrogate marker to differentiate antiinflammatory from neuroprotective activities. In such studies, a high frequency of MRI scans, as performed in the presently investigated clinical study, would facilitate the disentanglement of immunomodulatory-induced reductions in transient VV fluctuations from neuroprotective-induced reductions in BV losses that occur at different time scales. Distinguishing short-term processes from long-term VV and BV changes is vital, especially since the rate of VV expansion was shown to predict disease progression more strongly than the rate of whole brain atrophy (20).

The dynamics of CSF flow through the brain, and how this is altered under pathological conditions, is receiving increased attention in light of the recognition of the glymphatic system within the brain (37). CSF flows from the choroid plexus through the ventricular system and enters the brain parenchyma via the perivascular spaces along arteries, where it mixes with interstitial fluid (38). The CSF exits the brain along the perivenous spaces to the cervical lymphatics, the arachnoid granulations, and the meningeal lymphatics (39, 40). Immune cell accumulation in the perivascular space could interfere with normal glymphatic processes, and meningeal inflammation could disrupt clearance of the CSF. We observed that meningeal inflammation was extensive in EAE brains by d11 p.i. and could already be detected as early as d5 p.i. This is consistent with other reports that meningeal inflammation preceded inflammation in the parenchyma (41). A recent report described GBCA enhancements in the leptomeninges in myelin oligodendrocyte glycoprotein–immunized (MOG-immunized) C57BL/6J EAE mice, and these were especially prominent during the initial disease peak (42). In proteolipid protein–immunized (PLP-immunized) SJL/J EAE mice, we also observed GBCA leakage early during disease, as shown by reduced T_1_ values, particularly in leptomeningeal and cerebellar regions. Impaired CSF clearance was reported in EAE (43) and could impact VV changes. Leptomeningeal inflammation is frequently observed in MS and other neuroinflammatory conditions (44, 45). Impairment of CSF elimination associated with meningeal inflammation could contribute to transient VV changes in MS. Support for this notion comes from a recent study using the positron-emission tomography tracer ^11^C-PiB, which showed reduced CSF clearance in MS patients compared with healthy controls (46).

Inflammatory processes could interfere with the normal function of the choroid plexus, leading to altered CSF composition or CSF hypersecretion. Inflammation and disruption of the choroid plexus tissue architecture is seen in MS (47, 48), and there is compelling evidence that the choroid plexus is implicated in the very early stages of neuroinflammation. The choroid plexus mediates physiological immune surveillance of the CNS by entry of immune cells into the CSF circulation (1), and it is also a site for entry of pathological Th17 cells in EAE (49). We previously detected accumulation of very small superparamagnetic iron oxide particles (VSOP) in the choroid plexus (50) by MRI, confirmed by histology. Importantly, VSOP could be detected in the choroid plexus before the onset of clinical EAE signs and before the detection of histopathology in the rest of the brain, indicating early involvement of the choroid plexus in the disease process (51). Furthermore, in our previous study first demonstrating VV changes in EAE, we showed an alteration in the T_2_ relaxation of the CSF, suggesting an increase in the free water fraction (15), possibly as a result of altered composition or increased production of CSF due to choroid plexus inflammation.

Insights into the mechanisms of how inflammation can disrupt choroid plexus function have come from recent studies on experimental models of posthemorrhagic hydrocephalus (PHH) (52, 53). While PHH was previously attributed to primary impairments in CSF reabsorption, these recent studies demonstrate the contribution of CSF hypersecretion to the pathogenesis of PHH. In a model of intraventricular hemorrhage, PHH was shown to be the result of CSF hypersecretion by the choroid plexus. This was driven by a TLR4- and NF-κB–dependent production of proinflammatory cytokines that, in turn, upregulate Ste20-type stress kinase (SPAK); this kinase activates ion transporters in the choroid epithelium to increase CSF production (52). This could be reversed with drugs targeting these signaling pathways, leading to protection from the experimentally induced ventricle enlargement (52). Another group used a small molecule inhibitor of SPAK-dependent phosphorylation of ion transporters and were able to reverse CSF hypersecretion in experimental ischemic stroke (53). These studies demonstrate how inflammatory processes at the choroid plexus can result in expansion of the cerebral ventricles due to CSF overproduction in PHH and have implications beyond hydrocephalus and stroke to other acute and chronic inflammatory diseases (54).

Variations in VV and BV are known to be dependent on circadian rhythm (55) and hydration status (56–59). Interestingly, the activity of the glymphatic system is greatly increased during sleep. In the current study, we can exclude circadian rhythm and dehydration as contributing factors, since all patients were scanned consistently early in the morning and were instructed to eat normally and drink sufficiently. Nevertheless, potential effects of other physiological processes on VV cannot be entirely excluded. All patients in the study were treated with atorvastatin, and some also with IFN-β; therefore, potential confounding drug effects on VV cannot be excluded. Patients who experienced acute relapses were also treated with corticosteroids, and while the MRI and clinical measures in those patients were postponed by 4 weeks to avoid confounding effects on GBCA enhancement, potential effects of steroids on VV, though unlikely, cannot be entirely excluded. In addition to biological variation, differences in slice position between scans and other technical sources of variation often cannot be completely controlled (26, 60). Nevertheless, no significant differences between VV contracting and noncontracting patients were observed in terms of age, sex, the proportion also receiving IFN-β therapy, and number of acute relapses during the study period.

During the initial EAE disease peak, the VV increase was pronounced — up to double the original size in some animals. Regardless of the extent of the VV changes, all VV returned to normal values upon remission of clinical signs. We detected evidence of neurodegeneration with FJ staining, which was consistently associated with inflammatory lesions, particularly in the cerebellum and brainstem. Nevertheless, the magnitude of the FJ staining at this early time point was modest, consistent with other reports showing mild FJ staining at similar time points in the C57BL/6J MOG EAE model (61). Together with the fact that the VV expansion occurred at such an early phase in the disease and was reversible, even toward the end of the study when mice still showed clinical signs of neurological disease, it is highly unlikely that the initial VV changes were a consequence of profound neurodegeneration. Rather, it is more likely that the early VV changes were the result of fluid dysregulation associated with inflammatory processes at this phase of the disease. In contrast, neurodegenerative damage in EAE accumulates progressively over time (62).

The VV changes in the MS patients were less severe than in the EAE animals. A minority of patients (5 of 33) in our cohort showed increases in VV in the study period, with no contractions; indeed, this minority was responsible for the significant increase in VV seen at the whole group level. This is in line with conventional expectations that VV of MS patients should steadily increase over time — notwithstanding normal physiological fluctuations (30). The fact that a majority of patients we analyzed showed VV contractions, as well as expansions, beyond the range of variation of healthy subjects suggests that other processes are at work, in addition to neurodegeneration. Stratifying the MS cohort based on the plus or minus 6% threshold of normal monthly variance in VV (25) revealed that the patients with contracting ventricles appeared to be at an earlier stage of their disease. If transient VV fluctuations are associated with acute inflammatory activity, then it stands to reason that these fluctuations would be more prominent in RRMS, when inflammatory processes dominate, than SPMS — when acute inflammation is less prominent and neurodegenerative processes dominate. This is in line with a previous study that reported that, while PPMS and SPMS patients had larger VV at baseline, RRMS patients showed the greatest VV changes over 6 months (63). However, that study was not designed to detect month-to-month changes in VV during the study period; therefore, it might have underestimated the relevance of VV expansions and contractions. Interestingly, that study also showed that PPMS patients had the largest VV at the start of the observation period (63), which might suggest that neurodegenerative processes played a larger role in their disease, right from the onset. These speculations will require careful study of PPMS and SPMS patients, with serial MRI.

The clinical study that was the source of our MS patient data was designed as a time series with monthly MRI scans, including multiple parameters such as GBCA enhancement. This permits the use of time-series analysis in order to look for correlations between the timing of changes across multiple parameters — an approach that is under-used in MS research. The data set we examined can be considered as a short-time series (64), and given that the measurements took place over 1 year, there is no expectation of seasonality in the data, which can be a confounding feature of time series that extend over several years. Considering the short duration and the modest sample size, we adopted a conservative approach to the time-series analysis, with no attempt to draw causal interpretations or forecast outcomes. Rather, the objective was to determine whether there was any evidence indicating that changes in VV occurred at the same time (or shifted in time) as changes in the other MRI and clinical parameters measured.

The time-series analysis detected several significant cross-correlation coefficients distributed among the other MRI and clinical parameters. This revealed a complex picture, in which the timing of VV changes in some patients was significantly correlated with the timing of changes in other parameters. This suggests that VV changes may reflect disease activity, although no single parameter dominated the set of significant coefficients. We repeated the time-series analysis using CEL volume — the gold standard for MS disease activity — to see how the timing of changes in this parameter compared with the other parameters (including VV). There was a high degree of cross-correlation between CEL volume and CEL count at time lag 0, meaning that changes in CEL volume occurred at the same time as changes in CEL count, in some though not all patients. Aside from this expected cross-correlation, lesser significant coefficients were observed, and these too were distributed among all other parameters. This suggests that the degree to which the timing of changes in VV and CEL volume reflect changes in other disease-related parameters was comparable. In contrast, when the analysis was done to compare the timing of changes in performance in the HPT with other disease-related parameters, only 5 significant cross-correlation coefficients were retained after correction for multiple comparisons. This could reflect that the HPT measures motoric performance and may be related to spinal cord involvement (65, 66) or may simply be less indicative of rapid changes when compared with parameters such as VV and CEL volume.

The diagnostic value of GBCA for revealing disease activity in MS is indisputable. Nevertheless, there have recently been increasing safety concerns regarding the potential for long-term deposition of GBCA in the brain (9–12). In light of these concerns, there is a strong motivation to find contrast-free quantitative MRI parameters, which can serve as markers of disease activity. In this line, quantitative MRI measures such as T_1_ mapping, especially at ultra-high magnetic field strengths, can be especially informative (67). In the current study, we used T_1_ mapping of EAE brains to quantify gadolinium leakage as an indicator of BBB disruption. Crucially, we could show that the timing of VV changes coincided with the peak of BBB leakage and that both of these significantly preceded the onset of clinical signs of EAE. The T_1_ maps showed a pattern of contrast enhancement typical for SJL mice, with extensive involvement of the cerebellum, meninges and periventricular regions. This corresponded to histological detection of immune infiltrates and gliosis in these locations, and the severity of the histopathology correlated with the magnitude of VV changes. Thus, VV changes may be considered as a surrogate indicator of inflammatory activity. Future studies using even more sensitive histological methods for detecting BBB disruption would add further detail, complementing the quantitative T_1_-mapping approach.

In summary, close monitoring of VV changes in individual MS patients will be important to understand whether these are truly transient or persistent. Persistent changes would indicate irreversible progressive destruction, while transient events might be related to other pathological mechanisms superimposed upon the neurodegenerative process. Being able to disentangle these intermingled processes will have profound implications for determining more effective treatment strategies. Given that the RRMS patients with contracting VV in the current study appeared to be at an earlier phase of their disease, it is tempting to speculate that the cessation of VV volatility may be an indication of transition to the progressive phase of disease. This hypothesis remains to be confirmed in larger-scale data sets. Nevertheless, changes in VV could serve as a quantitative MRI biomarker of disease activity, which can be readily acquired in the course of routine clinical investigations of MS patients. However, in order for this to become part of routine clinical practice, it will be necessary to have tools available for the clinician to quickly and seamlessly integrate the calculation of VV into the clinical workflow. Continuing advances in machine learning–based methods for analysis of MRIs will be crucial for these developments. To accompany VV monitoring, further technical developments in MRI-based methods to monitor CSF flow (68) and composition (69) — as well as inflammatory processes (70) — are becoming increasingly available, particularly at higher magnetic field strengths. Together, these tools can help drive our understanding of the dynamic changes in the CSF compartment during neuroinflammatory disease and can lead to superior diagnosis, prognosis, and monitoring of patients.

## Methods

### Induction of EAE.

EAE was induced as previously described (15). Female SJL/J mice (8–12 weeks) were obtained from Janvier SAS. A total of *n* = 35 mice in 6 cohorts were used in the study. Mice were immunized s.c. with 250 μg PLP_139–151_ (Pepceuticals Ltd.) and 800 μg mycobacterium tuberculosis H37Ra (Difco), and they were administered 200 ng of pertussis toxin (List Biological Laboratories) on d0 and d2. Mice were weighed and scored daily as follows: 0, no disease; 1, tail weakness and righting reflex weakness; 2, paraparesis; 3, paraplegia; 4, paraplegia with forelimb weakness or paralysis; and 5, moribund or dead.

### Animal MRI.

Animals were anesthetized with a mixture of 1%–1.5% isofluorane (Abbott GmbH & Co.), air, and oxygen, and they were placed on an animal holder, kept warm with circulating water. Body temperature and respiration rate were monitored continuously with a remote monitoring system (Model 1025, SA Instruments Inc.). MRI was performed (Supplemental Methods), keeping slice positioning constant through the experiment, positioned parallel to the base of the brain. Mice were imaged at baseline, 2 days before immunization, and every 2–3 days after immunization, until day 64 after EAE induction. A total of *n* = 9 mice underwent the full longitudinal examination. We confirmed that the signal-to-noise and contrast-to-noise ratio (between cortex and ventricles) were consistent throughout the entire study period.

### Analysis of MRI data.

Quantification of mouse brain VV was done as in our previous study (15), using FMRIB Software Library (FSL v5.0, www.fmrib.ox.ac.uk/fsl) (71–73), and the transformed ventricle masks were manually corrected (Supplemental Methods). Data from the T_1_ maps were extracted and postprocessed using MATLAB (MathWorks Inc.), MATLAB Image J software module (MIJ), and Advanced Normalization Tools Toolkit (74, 75) (Supplemental Methods).

### Histology.

Brain tissue was collected from *n* = 19 mice at various time points: baseline (unimmunized controls) and d3, d5, d8, and d11 p.i. After terminal anesthesia using ketamine/xylazine, mice were perfused transcardially with 20 mL PBS and then with 20 mL zinc fixative (0.5% zinc acetate, 0.5% zinc chloride, 0.05% calcium acetate) (Sigma-Aldrich). Following extraction, brains were postfixed, frozen in OCT, and cut into 15 μm cryosections. Sections were stained with H&E according to standard procedures. H&E-stained tissue sections were reviewed and scored for the presence of inflammation in the following regions: meninges, periventricular regions, brainstem, cerebellum, cortex, and hippocampus. Absence of inflammation was scored as 0; minor signs of inflammation as +; multiple inflammatory foci as ++; and severe inflammation as +++. These semiquantitative scores were then correlated with the VVs and ΔT_1_ values. Immunostaining protocols (Supplemental Methods) were also performed.

### Healthy human controls.

Data from healthy control subjects were obtained from 2 publications that performed repeated serial MRI scans on healthy individuals and which had made their raw data available to the scientific community (25, 26). In one study to investigate intraindividual variability in brain structures and other measurements, subjects (*n* = 6) underwent multiple serial MRI scans over 6 months. From this data set, we selected scans at 1-month intervals in order to match the timing of the scans in the MS patient cohort. Demographic details of these subjects are listed in Supplemental Table 1. In a separate study to evaluate intra- and intersession reliability of brain imaging and automated segmentation software, *n* = 3 subjects underwent repeated MRI scans within a 31-day period (26). Given the retrospective nature of our study, it was not possible to age-match the healthy controls with the MS patient cohort. Both studies acquired T_1_-weighted scans, using 3.0 Tesla clinical MR scanners; details about the scan parameters can be found in the original publications (25, 26). Absolute VVs from scans of healthy controls were obtained using FreeSurfer v5.3 (76).

### MS Patient cohort.

Data from RRMS patients were obtained from our previous clinical study investigating oral atorvastatin therapy (NCT00616187) (24). All patients (*n* = 33) in this study had clinically definite MS (77) and had at least 1 CEL on brain MRI at the time of enrolment. Clinically active disease (i.e., signs of relapse) was not required for inclusion in the study. In addition to treatment with atorvastatin, 10 of 33 patients were cotreated with IFN-β. Patients who experienced acute relapses underwent corticosteroid treatment (methylprednisolone) for 3–5 days; subsequent MRI and clinical examination was postponed by 4 weeks to avoid confounding effects. Patients underwent 13 MRI examinations at 1-month intervals over the course of 1 year. T_1_- and T_2_-weighted MRI scans were acquired using a 1.5 Tesla clinical MR scanner (Siemens Sonata, Siemens Medical Systems). Diffusion-weighted imaging was performed, and the apparent diffusion coefficient (ADC) calculated in 4 regions of interest: corpus callosum (ADC CC), basal ganglia (ADC BG), brainstem (ADC BS), and normal-appearing white matter (ADC WM). All scans were performed between 8 and 9 a.m. in order to avoid circadian bias, and patients were advised to have regular meals and drinks to avoid dehydration. Detailed information on the scan parameters can be found in the original publication (24). Demographic and clinical details from the original publication are listed in Supplemental Table 1 (note that the current study used 33 patients who completed the full MRI scan protocol at all time points).

Analysis of MRI Data (Supplemental Methods) included VV and BV, CEL numbers and volumes, T_2_-weighted lesions and BH lesions. Additional parameters included the Paced Auditory Serial Addition Test (PASAT), the 9-hole peg test of the dominant (hpt dom) and nondominant (hpt ndom) hand, and the timed walk test (twt). EDSS scores were assessed at baseline and at selected time points during the study.

### Statistics.

For each patient, VVs, as well as lesion volumes, lesion counts, and additional clinical measures that were gathered and quantified from the 12-month clinical study, were analyzed using time-series analysis. The CCF calculates the cross-correlation coefficient, which indicates the relationship between 2 metrics as they change over time. Significant coefficients indicate that events of 1 series precede (negative time lag) or follow (positive time lag) the events of another series. Absence of significant CCF coefficients indicates that no temporal relationship between the 2 series exists. For this 12-month clinical study, we adopted a conservative approach, considering only those coefficients that were significant at time lags between –2 to +2 months. Given that the CCF requires continuous data, noncontinuous ordinal variables such as EDSS were not included in the time-series analysis. All time series for each individual patient for each parameter were detrended by first-order differencing, in which each point is replaced by the difference between its value and the value of the preceding point. This was done to ensure that the time series were stationary — i.e., lack an overall trend — in order to avoid spurious false-positive cross-correlations. The differencing was done using the R package forecast (78). Following first-order differencing, we confirmed stationarity for all time series for all patients with the Augmented Dickey-Fuller test (79), using the R package fUnitRoots (80).

The CCF was then applied using the R package time series (81) to look for temporal relationships between the VV time series and the time series for the other parameters for each individual patient. The Benjamini-Hochberg FDR method was used to correct for multiple comparisons, with FDR-corrected *P* < 0.05 considered significant. We also verified that there was no autocorrelation (periodicity) in the time series of each parameter we studied, using the ACF function (R package, time series).

Statistical analysis (including those in Supplemental Methods) was done using the statistical computing environment R v.3.3.4 (https://www.R-project.org) and GraphPad Prism v.5.01 (GraphPad Software, Inc.). All statistical tests are specified throughout the mansucript.

### Study approval.

Animal experiments were carried out in accordance with the guidelines approved by the Animal Welfare Department of the LAGeSo State Office of Health and Social Affairs in Berlin and in accordance with international guidelines on the reduction of discomfort (86/609/EEC). All procedures performed in studies involving human participants were in accordance with the ethical standards of the institutional and/or national research committee (Charité, German Federal Institute for Drugs and Medical Devices, BfArM) and with the 1964 Helsinki declaration and its later amendments. Written informed consent was received from participants before inclusion in the study.

## Author contributions

JMM, AS, LB, and SW acquired EAE data. JMM, PRD, JP, HMR, CP, AE, CID, HW, JBS, JW, MS, AP, and SW analyzed data. FP, SW, JW, JBS, and FZ conducted the clinical trial. JMM and SW wrote the manuscript. TN and FP critically reviewed the manuscript. SW, JMM, FP, and TN conceptualized the study.

## Supplementary Material

supplemental data

## Version 1 11/05/2020

Electronic publication

## Version 2 11/06/2020

Updated author list
