# Supplementary material for: Transient enlargement of brain ventricles during relapsing-remitting multiple sclerosis and experimental autoimmune encephalomyelitis
Source: JCI Insight. 2020 Nov 5;5(21):e140040. doi: 10.1172/jci.insight.140040 (PMC7710287; doi:10.1172/jci.insight.140040)
Supplement: supplemental data [file jciinsight-5-140040-s229.pdf]

Supplemental Data and Methods

Supplemental Figure 1

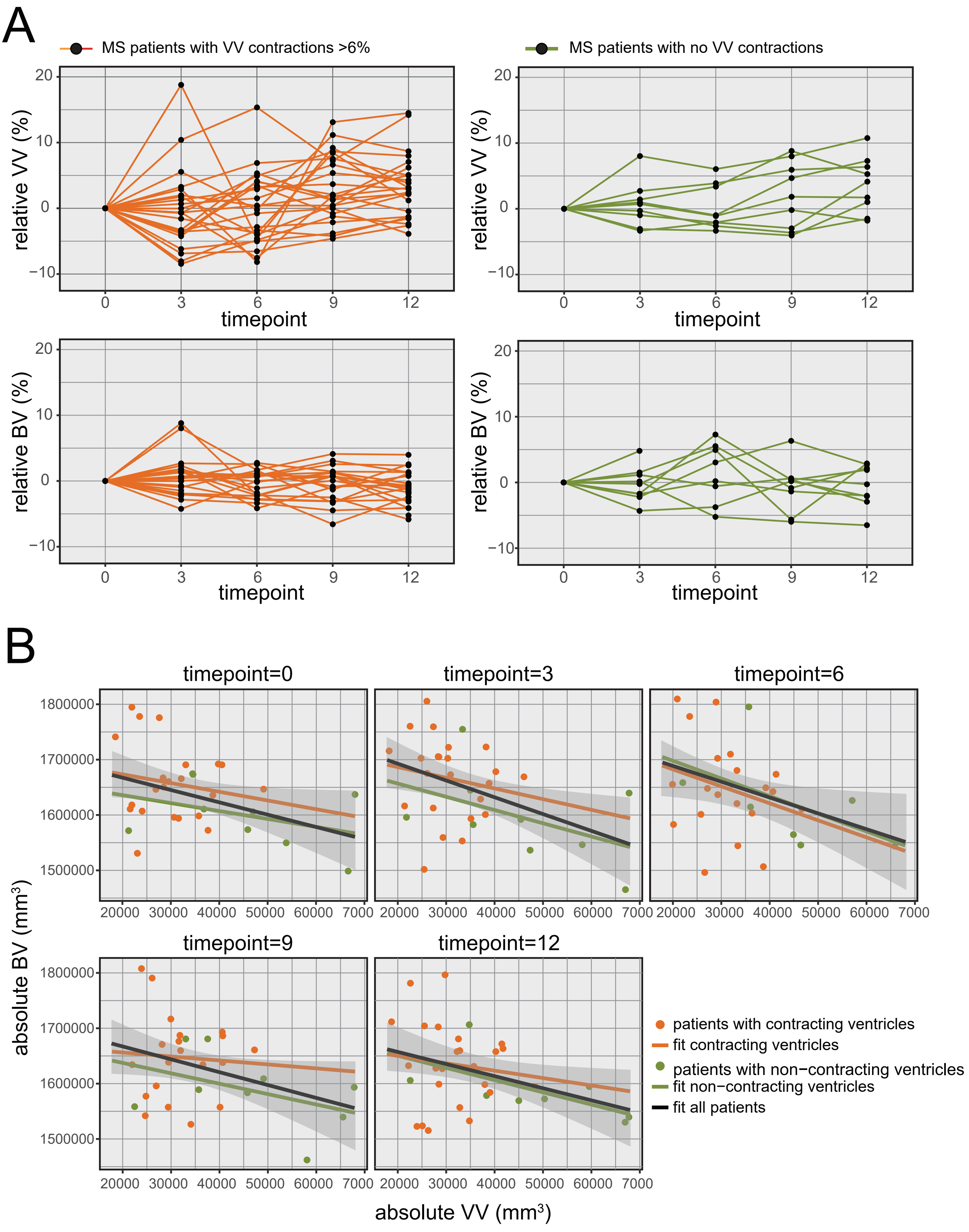

Supplemental Figure 1: *Correlations between ventricle volume and brain volume*. (**A**) Relative ventricle volume (VV) (upper panels) at timepoints 0, 3, 6, 9 and 12 months, for MS patients with contracting (left, orange) and non-contracting (right, green) VV (normalized to timepoint 0; data is the same as shown in Fig. 6B, C in the main manuscript). The corresponding brain volumes (BV) at these timepoints are shown in the lower panels. There was no statistically significant change in BV over time, considering either the contracting or non-contracting cohorts (p=0.875, p=0.977, respectively, 2-factor ANOVA) or the entire cohort (p=0.1167, Wilcoxon test). (**B**) There was a significant correlation between VV and BV at each of the investigated timepoints (fit with 95% confidence interval for the entire cohort, black; fit for the VV contracting and non-contracting MS patients, orange, green, respectively). The correlations were significant considering the entire cohort, but not the contracting and non-contracting patients separately (Pearson correlation coefficients and p-values are listed in Supplemental Table 2).

Supplemental Table 1

	Sex (m, f)	Age (yrs)	Duration of disease in months	Duration of IFN-β pre-treatment in months	EDSS at inclusion	Total no. of relapses since disease onset	No. of relapses 12 months prior to treatment
**All MS patients[a]**	20 / 21	35.4 (19-51)	84 (2-317)	n.a.	1.67 (1.4, 0-6)	4.07 (2.6, 1-12)	1.46 (1.1, 0-4)
**Patients w/ IFN-β[a]**	9 / 7	37.9 (24-48)	116.3 (26-317)	48 (7-115)	2.50 (1.5, 0-6)	5.75 (2.6, 3-12)	1.63 (1.3, 0-4)
**Patients w/o IFN-β[a]**	11 / 14	33.9 (19-51)	63.4 (2-229)	n.a.	1.14 (1.1, 0-4)	3 (2.1, 1-10)	1.4 (1.0, 0-4)
**Healthy controls[b]**	2/4	29 (24-32)	n.a.	n.a.	n.a.	n.a.	n.a.

Clinical and demographic baseline data of patients and healthy controls. Values are mean (standard deviation, range). Abbreviations: w/, w/o IFN-β: with / without interferon-beta pre-treatment / comedication.

[a] Modified from Paul et al., Oral high-dose atorvastatin treatment in relapsing-remitting multiple sclerosis. PloS one. 2008;3(4):e1928.

[b] taken from Filevich et al., Day2day: investigating daily variability of magnetic resonance imaging measures over half a year. BMC neuroscience. 2017;18(1):65.

Supplemental Table 2

timepoint	contracting patients[a]	non-contracting patients	all patients
0	R = -0.175, p = 0.4353	R = -0.384, p = 0.3068	R = -0.395, p = 0.0281
3	R = -0.179, p = 0.4145	R = -0.470, p = 0.2014	R = -0.452, p = 0.0093
6	R = -0.270, p = 0.2487	R = -0.583, p = 0.1291	R = -0.414, p = 0.0285
9	R = -0.066, p = 0.7822	R = -0.429, p = 0.2496	R = -0.363, p = 0.0531
12	R = -0.129, p = 0.5480	R = -0.6552, p = 0.0554	R = -0.368, p = 0.0352

[a] Pearson correlation R- and p-values for correlation between absolute brain volume and absolute ventricle volume

Supplemental Methods

MRI methods

MRI was performed on a 9.4 Tesla MR animal scanner (Biospec 94/20 USR, Bruker Biospin), using a birdcage radiofrequency (RF) coil tailored for mouse brain imaging and ideal for T_1 brain mapping (71). A RARE sequence was used for anatomical imaging (TR=3 sec, TE=14.3 ms, FOV=16.5x16.5 mm², matrix=512x512, number of slices=15, slice thickness=500 µm). Coronal MR images of the entire brain were acquired in 2 min 6 sec. The contrast agent gadolinium-diethylenetriamine-pentaacetate (300 nmol/g Gd-DTPA, Magnevist, Bayer-Schering) was infused via an intravenous cannula over 2 min, during each imaging session. A RARE sequence with variable repetition time (VTR) was used to generate T_1 maps pre- and post-contrast administration (RARE-VTR: TE=11.53 ms, matrix=128×85, slices/thickness=11/500 µm, VTR=0.38, 0.55, 0.94, 1.48, 2.40 and 7.00 sec, echo train length=4)

Analysis of mouse MRI data

In the mouse, tissues external to the brain were eliminated from MR images using the brain extraction tool (BET) of FSL. One mouse brain MR data set was used as reference for the whole study. A ventricle mask was generated from this data set using the FMRIB's Automated Segmentation Tool (FAST). Following this step, brain images from all time points for each individual mouse were registered to the reference image using the FMRIB Linear Image Registration Tool (FLIRT), to yield a transformation matrix. The transformation matrix for each individual was then applied to the ventricle mask, to determine the absolute ventricle volume in µl for each mouse at each time point. The transformed ventricle masks were overlaid against the original MR images, and anatomical errors in the segmentation were manually corrected, using the software ITK-SNAP (www.itksnap.org) (75), to yield corrected ventricle volumes. Consistency of the manual correction steps was confirmed by performing replicate corrections of randomly selected individuals and time points. Relative ventricle volume changes were calculated as the ratio of the volume at each time point to the pre-immunization volumes, and expressed as percent change.

Data from the T_1 maps was extracted and post-processed using MATLAB (The MathWorks, Inc.), MIJ and ANTs Toolkit (76, 77). T_1-weighted images (from the RARE-VTR with the longest TR) and T_1-maps were filtered using adaptive non-local means denoising (78). 3D intra-subject registration (rigid, affine, elastic) was done to register scans from each time point onto the first time point (pre-immunization). This was performed on the pre-contrast RARE-VTR T_1-mapping image with the longest TR, using a cross correlation similarity method. The warping fields were then applied to the T_1-maps. ROIs delineating the brain and cerebellum were manually defined for all slices from the first time point then applied to the entire series. The average T_1 relaxation time was computed for each

ROI and the corresponding delta-T_1 maps calculated as described (79). The delta-T_1 (difference between pre- and post-contrast T_1) was calculated and plotted over time.

Analysis of human MRI data

Quantification of ventricle volumes (VV) and brain volumes (BV) for the MS patient cohort was done using the FSL v5.0, for each time point and each individual patient. Tissues external to the brain were removed from the T_1-weighted images using the Brain Extraction Tool (BET). T_1-weighted images were registered onto T_2-weighted images using FMRIB's Linear Image Registration Tool (FLIRT), and subsequently registered to the MNI standard brain, from which a ventricle mask was applied. Ventricle segmentation masks were then generated from the T_2-weighted images. The raw segmentations were overlaid with the original MR images and manually corrected by experienced raters in ITK-SNAP to yield absolute ventricle volumes. Relative ventricle volume changes were calculated as the ratio of the volume at each time point to the baseline volumes, and expressed as percent change.

Numbers and volumes of CEL, T_2-weighted lesions and black hole (BH) lesions were quantified using the MedX v.3.4.3 software package by experienced raters. The total burden of lesion volume during the study was calculated as the area under the curve of the plot of lesion volume across all time points.

Immunohistological staining

Cryosections were dried for 30 min at room temperature, washed in 0.1M PBS, and incubated with protein blocking solution (0.5 % triton + 10 % normal goat / rabbit serum), and avidin-biotin blocking, as needed. Sections were then incubated overnight at 4°C with primary antibodies against GFAP (rabbit anti-mouse, 1:500, Abcam), CD3 (polyclonal rabbit anti-human, 1:500, DAKO), and F4/80 (biotin rat anti-mouse, 1:200, Abcam) followed by goat anti-rabbit-Alexa488 (1:500, Invitrogen) or streptavidin-Pe-Cy5 (1:500, Invitrogen), and subsequently stained with the nuclear stain DAPI. Images were acquired on a fluorescence microscope (Olympus BX-51). For Fluoro-Jade staining, sections were incubated in 0.06 % potassium permanganate for 20 min at room temperature, then rinsed in distilled water. The sections were then incubated in a solution of 0.001 % Fluoro-Jade B (Chemicon) in 0.1 % acetic acid for 20 min at room temperature in the dark, then rinsed 3 times in distilled water and subsequently stained with DAPI. Fluoro-Jade stained slides were scanned using a Panoramic MIDI II slide scanner (3D Histech). Images were visualized using Case Viewer v.2.3 (3D Histech). Obvious tissue processing artifacts were manually removed, and intensity of Fluoro-Jade staining quantified in the whole section using ImageJ v1.51 (NIH).

Other statistical analysis. Additional comparisons of two or more groups was done using Student's t-test or ANOVA, or non-parametric Mann-Whitney, Wilcoxon or Kruskal-Wallis tests, as required. Kaplan-Meier survival curves were analyzed using the logrank test with Bonferroni correction for multiple comparisons. The non-parametric Spearman correlation was used to analyze histopathology scores. All tests were 2-sided. p-values less than 0.05 were considered significant. Data analysis was done using the statistical computing environment R v.3.3.4 (https://www.R-project.org) and GraphPad Prism v.5.01 (GraphPad Software, Inc.).
